# Leveraging artificial intelligence to optimize neuromodulation in the treatment of patients with chronic pain

**DOI:** 10.3389/fpain.2026.1827351

**Published:** 2026-05-29

**Authors:** Rohail Siddiqi, Abdullah Cheema, Morad Marikh, Amir Alsaidi, Lisiane Pruinelli, Saba Javed, Salahadin Abdi

**Affiliations:** 1 Paul L. Foster School of Medicine, Texas Tech Health Science Center, El Paso, TX, United States; 2 Department of Family, Community and Health Systems Science, University of Florida, Gainesville, FL, United States; 3 Department of Surgery, University of Florida, Gainesville, FL, United States; 4 Division of Anesthesiology, Critical Care and Pain Medicine, Department of Pain Medicine, The University of Texas MD Anderson Cancer Center, Houston, TX, United States

**Keywords:** artificial intelligence, chronic pain, machine learning, neuromodulation, patient selection, personalized medicine, predictive modeling, spinal cord stimulation

## Abstract

Chronic pain that persists despite conventional therapy remains a major clinical and economic burden. Spinal cord stimulation (SCS) offers an alternative for treatment of refractory pain, yet about 30% of patients experience limited or no benefit due to the subjective nature of current patient selection processes. In this mini review, we explore how artificial intelligence (AI) and machine learning (ML) can optimize patient selection for and improve outcomes of SCS. Existing approaches to patient selection rely on patient-reported outcomes and physician judgment, which fail to capture multifactorial influences like clinical, psychological, and socioeconomic factors that determine success of pain treatment. AI and ML methods, including supervised learning, feature selection, and risk stratification, can analyze complex datasets from electronic health records to identify predictive variables associated with sustained pain relief. Integrating these technologies into the patient selection process may enhance precision in patient screening, reduce SCS device failure and explantation rates, and lower healthcare costs. Furthermore, adaptive learning algorithms can refine predictive models in real time, improving accuracy as new data emerge. With transition from subjective assessment to data-driven decision-making, AI-guided strategies have the potential to make SCS a more reliable, equitable, and cost-effective therapy for chronic pain management.

## 1 Introduction

Chronic pain remains one of the most challenging conditions to manage, often requiring multimodal and long-term therapeutic strategies. Spinal cord stimulation (SCS) is an implantable neuromodulation therapy designed to alleviate refractory pain when conventional treatments fail (1). Although SCS has improved the quality of life for many patients, clinical outcomes vary substantially across individuals. Determining which patients will experience sustained benefit remains a major clinical challenge.

The current SCS trial protocol, mandated by the FDA, involves the use of an external stimulator for several days to evaluate short-term pain relief before permanent implantation of SCS device (2). Despite this precaution, failure rates of up to 30% have been reported (3), reflecting huge variability in therapeutic success and the limitations of subjective assessment of pain- reported pain outcomes during short-term, non-blinded trial stimulation used to determine eligibility for permanent SCS implantation. These failure rates indicate both emotional and economic burdens on patients and the healthcare system.

The variability in SCS failure rates demonstrates the need for more objective and data-driven patient selection criteria. Present screening methods rely heavily on subjective pain reports, physician judgment, and limited psychometric evaluations (4). However, these factors alone inadequately predict long-term success. In contrast, artificial intelligence (AI) and machine learning (ML) approaches are well suited to analyze large, heterogeneous datasets and reveal nonlinear relationships that traditional methods may overlook (5).

AI encompasses computational techniques that simulate human cognitive functions, learning, reasoning, and pattern recognition while operating at scales far beyond human capability. Within healthcare, AI has demonstrated an expanding role in detecting hidden trends across multidimensional patient data, improving diagnostic accuracy, and guiding personalized interventions (6, 7). Integrating demographic, psychological, and socioeconomic data into AI-driven analyses could transform how candidates for neuromodulation therapies are identified.

In the context of SCS, AI-assisted patient selection offers the opportunity to reduce implant failure rates, improve quality of life, and optimize healthcare resource allocation. Leveraging this technology may refine risk stratification and enable use of adaptive feedback systems that continuously learn from patient outcomes (8).

Despite growing enthusiasm for AI-guided neuromodulation, current literature remains fragmented, with limited integration of clinical, psychophysical, and socioeconomic datasets into unified predictive frameworks. Furthermore, most studies remain retrospective, single-center analyses lacking external validation. This review aims to synthesize emerging evidence while proposing conceptual models for multidimensional data integration to guide precision neuromodulation.

**Figure 1:**
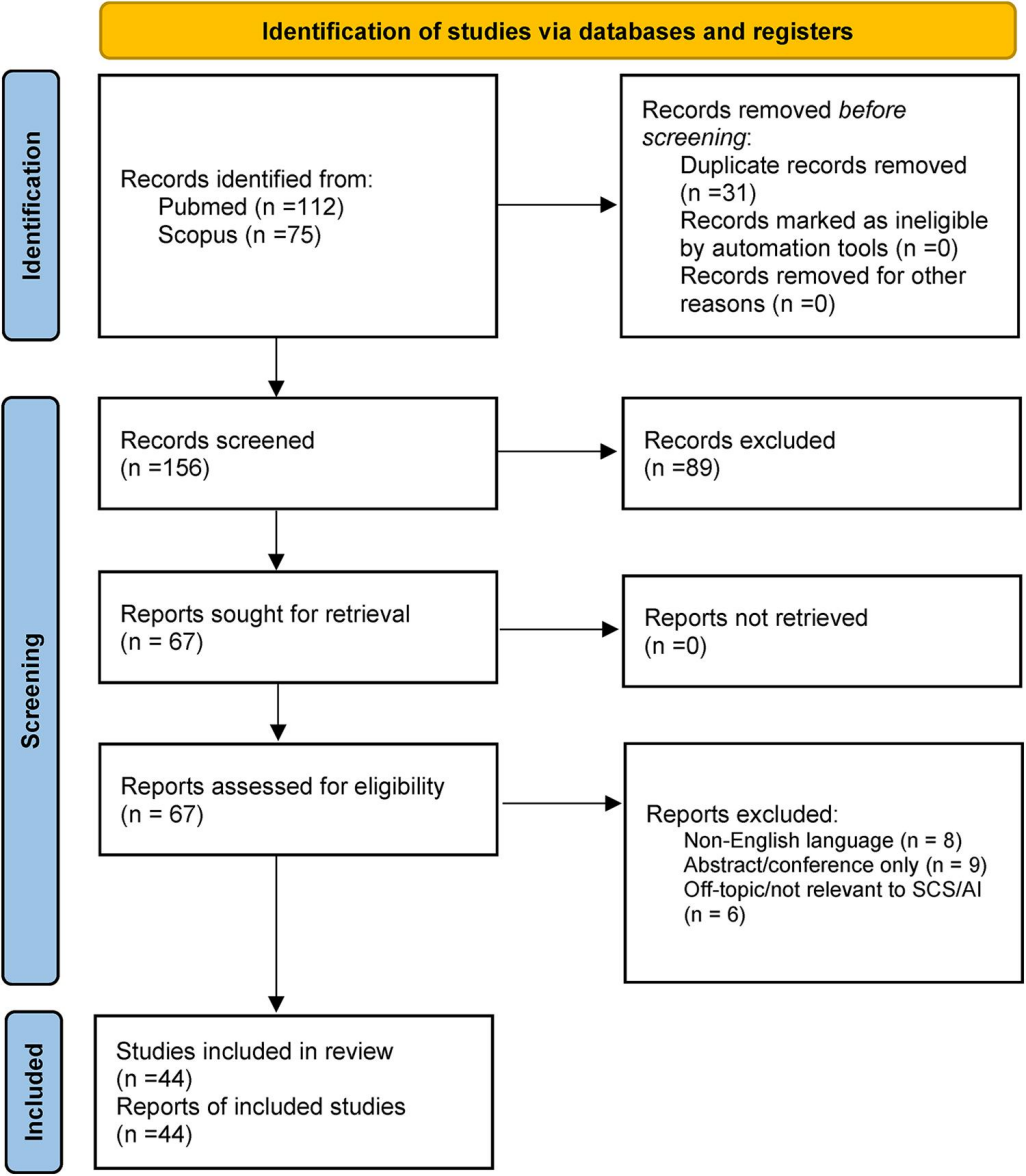
PRISMA flow diagram illustrating the literature search and screening process. Records were identified from PubMed and Scopus (2010–2026) using terms related to spinal cord stimulation, artificial intelligence, machine learning, chronic pain, patient selection, and predictive modeling.

A literature search was conducted by the first author using PubMed and Scopus to identify relevant studies published between 2010 and 2026. The following search terms were used: “spinal cord stimulation,” “neuromodulation,” “artificial intelligence,” “machine learning,” “chronic pain,” “patient selection,” “outcome prediction,” “predictive modeling,” and “risk stratification,” applied individually and in combination. Articles were included if they were published in English, addressed SCS outcomes or AI/ML applications in pain management, and were available as full-text publications. Review articles, clinical trials, retrospective studies, and consensus guidelines were all considered. Non-English publications, conference abstracts, and studies unrelated to neuromodulation or AI were excluded. Reference lists of included articles were also reviewed to identify additional relevant sources. A total of 44 articles met inclusion criteria and were incorporated into this review. The literature screening process is summarized in Figure 1.

## 2 Current status and recent outcomes of SCS

SCS has long served as an important treatment option for patients with chronic pain who have not experienced relief through conventional management. Despite its advantages, the reported success rates for SCS vary widely, and a significant proportion of patients experience suboptimal outcomes (9). Clinical success is typically defined as at least a 50% reduction in pain intensity, improved functional ability, and enhanced quality of life. However, these outcomes differ across patient populations, and predicting long-term benefits remains difficult.

About 30% of SCS implantations fail to deliver satisfactory results, reflecting the influence of five key factors that contribute to variability in response (Table 1).

**Table 1:** Summary of studies evaluating the cost-effectiveness of SCS compared with CMM.

Reference	Study objective	Major findings	Cost-effective
Kumar (45)	Evaluate cost-effectiveness of SCS vs. CMM	SCS is more cost-effective vs. CMM with ICER [CAD $9,293.00 (US $6,717.23) per QALY]	Yes
Hornberger (9)	Measure lifetime cost differences between SCS systems	Rechargeable systems save up to $100,000 over time	Yes
Manca (46)	Assess HRQoL and cost of SCS vs. CMM for neuropathic pain	SCS improves HRQoL compared with CMM but incurs higher costs at 6 months	Yes
Taylor (47)	Evaluate SCS vs. CMM for FBSS	SCS reduces pain and overall healthcare costs over time	Yes

FBSS, failed back surgery syndrome; HRQoL, health-related quality of life; ICER, Institute for Clinical and Economic Review; QALY, quality-adjusted life-year.

### 2.1 Patient selection

Current patient selection criteria for SCS often rely on patient-reported outcomes and subjective pain assessments, which are inherently inconsistent (10). Objective predictive tools are limited, and standardized metrics to identify patients most likely to benefit from SCS are still lacking. Psychophysical and neurophysiological testing such as nerve conduction studies and quantitative sensory testing could offer a more data-driven framework for patient evaluation but remain underutilized (11). Recent expert consensus guidelines for spinal cord stimulation emphasize the urgent need for improved patient-selection methods to enhance the therapeutic success of SCS (12, 47).

### 2.2 Technical issues

Technical complications such as lead migration, hardware failure, and insufficient paresthesia coverage can reduce the efficacy of SCS (13, 14). Many centers still employ older SCS systems that lack features like closed-loop feedback and 3D navigation for precise lead placement. These technological limitations may increase SCS failure rates by reducing stimulation accuracy and responsiveness to the spinal cord due to physiological changes (15).

### 2.3 Psychological factors

Psychological conditions such as depression and anxiety can negatively influence pain perception and treatment outcomes. Comprehensive psychological screening and support are therefore essential components of successful SCS programs (16). However, ongoing psychological support and re-evaluation of mental health are often lacking. Structured psychological interventions that address the dynamic interaction between mental health and pain could improve adherence and overall response to SCS (17).

### 2.4 Clinical factors

The underlying etiology and duration of pain as well as prior interventions affect SCS outcomes. For example, responses of patients with failed back surgery syndrome or complex regional pain syndrome may differ from those of individuals with other pain conditions (18). Current SCS protocols often apply a generalized approach to patient programming and treatment delivery that fails to consider variables such as pain chronicity, surgical history, and mixed pain mechanisms (19). Tailoring SCS parameters to these clinical factors could enhance individualized care.

### 2.5 Socioeconomic factors

Socioeconomic disparities also play a role in patient access and treatment success for SCS. Individuals from lower income backgrounds may face limited insurance coverage or restricted access to specialized centers, leading to reduced SCS availability (20).

Furthermore, patient education and awareness about SCS options vary widely. Addressing these inequities through targeted education and policy interventions can improve outcomes and ensure equitable care delivery.

Collectively, these factors demonstrate that the variability in SCS outcomes has intersecting clinical, psychological, technical, and socioeconomic determinants. Current screening and follow-up methods often fail to capture this complexity. Integrating AI-driven analytical approaches may enable more objective assessment of patient-specific predictors of SCS response and personalized treatment planning, potentially reducing SCS failure rates and enhancing long-term pain relief.

## 3 Cost-effectiveness analysis of SCS

SCS can significantly improve quality of life for patients with chronic, refractory pain. However, when the therapy fails, the consequences extend beyond persistent pain, as patients also face increased financial, psychological, and functional burdens. In this section, we review evidence on the economic and quality-of-life implications of failed SCS and highlight the potential role of AI-driven strategies in reducing associated costs of SCS failure and explantation.

### 3.1 Expenditures when SCS fails

The financial toll of SCS is substantial for both patients and the healthcare system. Initial expenses, which comprise the implantable device, surgical procedure, and postoperative care, typically total about $30,000. This does not include the costs of subsequent complications or follow-up interventions. When the therapy fails, these costs escalate further due to performance of revision surgeries and explanations and the need for alternative pain management strategies.

Patients undergoing SCS device explantation have been shown to incur markedly higher healthcare costs than those who retain their devices (21) found that, in the year before implantation, explantation recipients had a higher median cost of total healthcare expenditures ($42,140 vs. $27,822), more frequent pain-related visits (180 vs. 103), and greater pain management expenses ($15,447 vs. $9,228). After implantation, their costs decreased more slowly (4% vs. 6%), and by the time of explantation, their total expenses were more than double than those for patients with maintained functioning systems.

Failed SCS often necessitates multiple revision surgeries, each compounding total healthcare expenditures (22). When explanation becomes unavoidable, additional surgical risks such as cerebrospinal fluid leakage, infection, and prolonged recovery further contribute to patient morbidity and healthcare system resource utilization and costs, including surgical, inpatient, and postoperative services.

### 3.2 Impact of SCS failure on patient quality of life

Beyond the monetary burden, failed SCS profoundly affects quality of life. The therapy is designed to reduce chronic pain and restore function; when it fails, pain typically recurs or worsens, leading to greater disability and loss of independence. Patients frequently report heightened psychological distress, including anxiety and depression, as pain relief expectations remain unmet (23).

Functional decline can also lead to diminished productivity and social withdrawal. Persistent pain often limits employment, reduces participation in daily activities, and hurts interpersonal relationships, ultimately worsening both mental and socioeconomic well-being (24).

These cascading effects highlight that SCS failure is not solely a procedural issue but rather a multidimensional health concern with significant consequences for patients' physical, emotional, and financial states.

## 4 Comparative cost-effectiveness of SCS vs. conventional therapies

Several studies have compared SCS with conventional medical management (CMM) in terms of both healthcare expenditures and patient-reported outcomes. Collectively, these analyses demonstrated that whereas SCS entails higher upfront costs, it is often cost-effective over time when successful, improving pain control, reducing reliance on medications, and enhancing quality of life (25).

## 5 Implications of SCS for AI-enhanced patient selection

Economic and clinical outcomes of SCS are tightly intertwined: every failed implantation represents not only a therapeutic setback but also a significant financial burden. By identifying patients most likely to have positive responses to SCS, AI-driven predictive models could substantially reduce explantation rates and unnecessary expenditures. Such models could also guide clinicians toward cost-effective strategies for SCS patient selection and treatment optimization by integrating multidimensional variables such as clinical, psychological, and socioeconomic factors into patient-selection algorithms.

Ultimately, optimizing patient selection with AI may improve both cost-efficiency and quality of life for individuals undergoing SCS, transforming neuromodulation into a more sustainable therapeutic option within modern pain management.

## 6 AI for precision patient selection and outcome prediction (ML approaches for enhancing SCS efficacy)

The integration of AI and ML into healthcare has opened new opportunities for personalized medicine, including applications in SCS for chronic pain management (26). By improving patient selection and enhancing the accuracy of outcome predictions, these techniques can enhance the overall efficacy and efficiency of SCS. In this section, we review key developments demonstrating how AI can optimize neuromodulation outcomes through precision modeling and continuous learning.

### 6.1 The need for precision in patient selection for SCS

Traditional patient selection for SCS relies heavily on short-term trial periods and subjective pain assessments. Although these approaches can offer temporary insights into short-term analgesic responsiveness and tolerability, they often fail to predict long-term therapeutic success (12).

Roughly 30% of implantations yield suboptimal pain relief, emphasizing the need for objective, data-driven predictive criteria. AI and ML systems are uniquely suited to capture the intricate interactions among biological, psychological, and environmental factors that determine treatment outcomes.

### 6.2 ML in healthcare

ML, a subset of AI, employs algorithms that learn from data to make predictions or classifications (27). In clinical contexts, ML excels at analyzing large, complex datasets and identifying subtle, multidimensional patterns that may elude human observation (28). This capability is especially valuable for patients with chronic pain, whose outcomes are shaped by numerous interacting variables such as pain duration, mental health status, and treatment history (29). By uncovering these multifactorial relationships across diverse inputs, ranging from genetics and comorbidities to lifestyle and psychosocial variables, ML can refine diagnostic accuracy, forecast therapeutic responses, and personalize treatment plans. Moreover, ML models can evolve dynamically as new data are incorporated, continuously improving predictive performance (6, 30). Beyond structured ML algorithms, broader AI-driven approaches are increasingly being explored to personalize chronic pain management by integrating patient-specific biological, psychological, and contextual variables into dynamic treatment frameworks (31).

### 6.3 Enhancing SCS efficacy with AI

#### 6.3.1 Data integration and analysis

(a) AI systems can combine diverse patient information, demographics, medical history, psychological profiles, and socioeconomic factors often available within electronic health records (32). Through multivariate analysis, ML models can uncover complex nonlinear relationships between these factors and SCS success rates (33). For instance, algorithms may reveal that specific combinations of prior treatments and psychological traits strongly predict long-term improvement in pain intensity and functional outcomes (34).
(b) Advanced feature-selection methods, including recursive feature elimination and Lasso regression, identify the most influential predictors of SCS treatment response while minimizing noise, thereby enhancing both ML model accuracy and interpretability (35, 36). Such feature-focused models clarify which patient characteristics, pain patterns, or prior interventions best forecast successful outcomes of long-term SCS therapy (34). Incorporating socioeconomic data into predictive modeling frameworks can also reveal disparities in access to SCS evaluation and therapy, enabling more equitable, targeted care (37).
(c) Recent studies have further demonstrated the practical application of these AI-driven approaches in clinical settings. Gopal et al. applied ML to intraoperative EEG data recorded during SCS surgery, combining principal component analysis and recursive feature elimination with patient-reported outcome measures to construct predictive models of SCS response, revealing novel neurophysiological markers of chronic pain that may serve as objective biomarkers for patient selection (38). Complementing this, Prunskis et al. conducted a comprehensive review demonstrating that integrating AI into SCS care, from predictive modeling for patient selection to real-time adaptive stimulation parameter adjustment significantly improves patient outcomes and reduces suboptimal responses (39).

#### 6.3.2 Predictive modeling

(a) Supervised learning techniques enable algorithms to “learn” from labeled datasets such as known successful vs. unsuccessful SCS cases and apply this knowledge to prediction of outcomes of SCS therapy among new candidates for spinal cord stimulation (34). Decision trees and support vector machines are highlighted here because they represent complementary supervised approaches that balance clinical interpretability with the ability to model complex, nonlinear relationships in heterogeneous patient data.
  (i) **Decision trees** split datasets into branches based on clinical and psychosocial variables, providing interpretable pathways such as early stratification by pain chronicity, followed by prior surgical history and psychological risk factors that highlight which patient traits (e.g., pain duration, prior surgery, anxiety level) most influence therapeutic success (40).
  (ii) **Support vector machines** define optimal boundaries between outcome groups within high-dimensional data. This is particularly valuable when outcomes depend on complex variable interactions, for example, when moderate anxiety predicts poor results of long-term SCS response only when pain duration is prolonged (34).
(b) These algorithms can transform subjective screening into quantifiable, evidence-based prediction of SCS efficacy. More recently, large language models such as GPT-4 have been evaluated as clinical decision-support tools for SCS candidate selection, demonstrating agreement with expert multidisciplinary teams and suggesting a potential role for AI in streamlining the screening process (41).

#### 6.3.3 Risk stratification

AI models can classify patients according to their likelihood of experiencing meaningful pain relief, a process known as risk stratification. By identifying high-risk individuals early, clinicians can tailor their pain management, such as offering additional psychological support or alternative interventions to improve success rates (42). Risk stratification also streamlines healthcare utilization by allocating resources to candidates most likely to benefit, thus optimizing both clinical outcomes and system-level efficiency.

#### 6.3.4 Continuous learning and adaptive algorithms

A defining advantage of AI systems lies in their capacity for continuous improvement. Through feedback loops, adaptive models incorporate new clinical data and outcomes to recalibrate predictive accuracy over time (43). For instance, if emerging evidence demonstrates that certain psychological factors significantly influence response to stimulation, an adaptive model updates accordingly, enhancing precision in patient selection and long-term reliability of outcome predictions.

Integrating AI and ML into SCS can transform neuromodulation into a more precise, cost-efficient, and patient-centered treatment. By identifying patterns of complex, multidimensional relationships invisible to traditional analytics, these systems can refine candidate selection, improve outcome prediction, and promote adaptive learning within clinical workflows. Ultimately, AI-enhanced decision support could increase SCS success rates, reduce healthcare expenditures, and deliver more equitable and personalized pain-management solutions.

## 7 Conclusion and future directions

Integrating AI and ML into SCS presents a transformative opportunity to improve patient selection, treatment outcomes, and healthcare efficiency. Traditional SCS candidate-screening methods depend largely on subjective reports of pain relief during short-term trial periods. This approach often fails to capture the multifaceted biological, psychological, and socioeconomic factors that influence long-term success of SCS, contributing to high variability in clinical outcomes and costs. In contrast, AI and ML offer a data-driven, objective framework for decision-making. By analyzing complex datasets that include clinical variables, psychosocial measures, and treatment histories, these systems can identify key predictors of therapeutic success that might otherwise go unnoticed. Tools such as feature selection, supervised learning, ensemble modeling, and risk stratification enable the creation of predictive models that enhance predictive accuracy and clinical interpretability. Moreover, the use of adaptive, continuously learning algorithms ensures that predictive models evolve in real time as new evidence emerges. This capacity for self-improvement can refine treatment personalization, guide SCS implant decisions, and inform postoperative management to further reduce the risk of failure.

Looking ahead, future studies of AI-guided SCS patient selection and outcome prediction should focus on developing validated, clinically deployable predictive models that integrate data from multiple sources, such as electronic health records, wearable devices, and imaging tools.

Collaborative efforts among clinicians, data scientists, and engineers will be crucial to ensuring that these systems are both interpretable and ethically implemented. Standardized protocols for AI training, validation, and transparency will also be essential to ensure patient safety and regulatory compliance. Notably, the American Society of Pain and Neuroscience has begun issuing formal guidelines for AI use in interventional pain practice, signaling growing institutional recognition of these technologies and the need for standardized frameworks governing their clinical deployment (44).

By combining data-driven analytics with clinical expertise, AI and ML can transform SCS from a trial-and-error procedure into a precision-guided therapy. This shift promises to not only enhance clinical outcomes but also reduce costs, improve equity in pain management, and ultimately improve the quality of life for individuals living with chronic pain.
